# Prosthetic Arteriovenous Graft Contact Dermatitis Masquerading as an Arteriovenous Graft Infection in a Hemodialysis Patient

**DOI:** 10.1177/2324709616658311

**Published:** 2016-07-08

**Authors:** Nagadarshini Ramagiri-Vinod, Hassan Tahir, Sandhya Narukonda, Medha Joshi

**Affiliations:** 1 Temple University/Conemaugh Memorial Medical Center, Johnstown, PA, USA

**Keywords:** AV Prostehtic Graft infection, Contact Dermatitis

## Abstract

Prosthetic arteriovenous (AV) graft is the second most common vascular access of choice in hemodialysis patients. Rare complications of such grafts are increasingly seen due to rising population of patients on hemodialysis. Infections and thrombosis are the most common complications. Though metallic implants are known to cause hypersensitivity skin reactions, prosthetic AV grafts are rarely known to cause such kind of reactions due to inert nature of materials used in their preparation. We present a case of 54-year-old male who developed contact dermatitis after AV graft creation which was mistreated initially as infection.

## Introduction

Contact dermatitis remains one of the leading skin diseases in the United States. Patients on hemodialysis are sensitized to many allergens, making them a potential subject for delayed type of hypersensitivity reaction over time. It is important to differentiate contact dermatitis from other diseases common in patients on hemodialysis such as cellulitis, drug-induced bullous reaction, tinea corporis, and drug-induced photosensitivity reactions.^1^ Prosthetic arteriovenous (AV) grafts, though considered inert, are not without complications.

## Case Description

**Figure 1.**
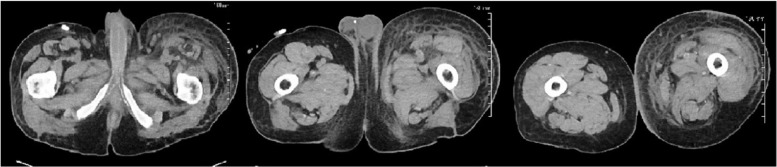
Computed tomography scan of pelvis without contrast showed extensive edema and congestion of the subcutaneous fat extending from the left external oblique musculature inferiorly along the lateral aspect of the pelvis into the upper left lower extremity.

**Figure 2.**
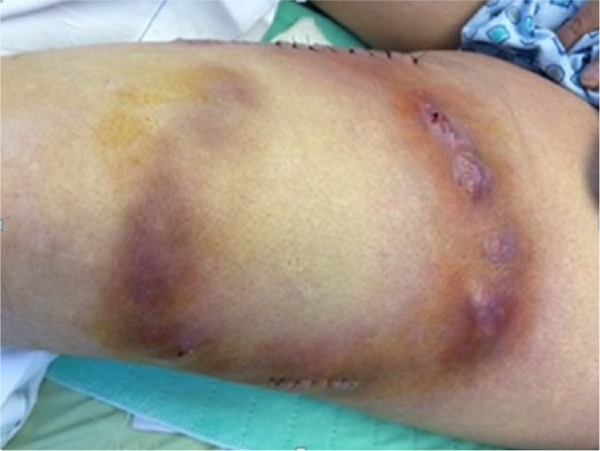
Erythema, swelling, and pustules at the site AV graft surgical site indicating contact dermatitis.

A 54-year-old male with past medical history of end-stage renal disease on hemodialysis, hepatitis C, ischemic stroke, depression, and anxiety was brought to the emergency department with the complaints of fever (100.2°F) and increased surgical site pain in left thigh. He underwent creation of a left superficial femoral artery to greater saphenous vein prosthetic AV graft 1 day prior to admission. Few hours after the procedure, he started having increased surgical site pain, erythema, and small vesicles at the margins of wound. On examination, the left thigh AV fistula site staples were intact. There was mild erythema, tenderness, and vesicles at margins. Serous discharge was noted from vesicles but no purulent drainage from wound area. Lab investigations showed creatinine level of 12.6 mg/dL and a potassium level of 6.4 mEq/L at the time of admission prompting hemodialysis. On admission, his vitals were normal and white blood cell (WBC) count was 10 000/cumm. The next day, his left thigh wound pain and swelling worsened concomitant with erythema, tenderness, and purulent discharge. Blood cultures were taken and drainage sample was sent for culture and sensitivity. His WBC count increased to 13 000 and erythrocyte sedimentation rate was 45; therefore, on suspicion of AV graft infection he was started on vancomycin 1.25 g intravenously given after each hemodialysis session. The vancomycin trough level was therapeutic, and blood cultures and drainage culture were negative. Despite the antibiotic therapy, his symptoms worsened with each passing day. Repeat blood and drainage cultures were consistently negative. Computed tomography scan of pelvis showed extensive edema and congestion of the subcutaneous fat extending from the left external oblique musculature inferiorly along the lateral aspect of the pelvis into the left upper extremity (Figure 1). Because of worsening symptoms, he underwent exploration of left thigh AV graft and samples were collected from perigraft tissue and perigraft fluid for culture and sensitivity. Once again, all cultures came back negative. Over the next couple of days, he started having purulent drainage from multiple locations along the skin overlying the graft (Figure 2). The decision was then taken to surgically remove the graft. The drainage sample from excised graft was also negative for bacteria. After graft removal, his symptoms markedly improved with complete resolution of symptoms in 7 days. In the meantime, he was given topical emollients for symptom relief only. Due to abrupt onset of symptoms within hours after AV graft placement, the diagnosis of AV graft contact dermatitis was made.

## Discussion

Arteriovenous graft is a surgically created anastomosis between an artery and vein via prosthetic conduit. The conduit can be straight or looped and placed superficially under skin for easy cannulation.^2^ AV fistulas are considered vascular accesses of choice in hemodialysis patients due to low morbidity and mortality and AV grafts are only second in option in terms of preference. However, old age, AV fistula primary failure, and inadequate maturation have resulted in increasing use of AV grafts in recent years.^3^ Prosthetic grafts are classified as biological and synthetic. Most commonly used material for making AV fistula are polytetrafluorethylene, Dacron, silicon, and polyurethane. Polytetrafluoroethylene (PTFE) grafts are preferred over biological and other synthetic grafts due to low thrombosis risk, longer patency, ease of implantation, and low risk of disintegration with infection.^4^ Major complications of AV graft include thrombosis and infections. AV fistula graft infection is more common than fistula infection affecting 9% to 20% of grafts.^5^ Infection is more common in thigh grafts, immunodeficiency, obesity, diabetics, and multiple surgical revisions.^3,5^ Graft infection can present in multiple ways: septicemia, abscess, draining sinus, pyrexia of unknown origin, erythema at cannulation site, hemorrhage, and pain at the dialysis site. Not uncommonly, graft infection may present atypically with no physical signs. Management includes intravenous antibiotics in all patients and total, subtotal, or partial graft excision depending on patient condition and extent of infection.^6^

Cutaneous reaction is a known complication of metallic implants presenting most commonly as eczematous skin reaction, though urticarial, bullous, and vasculitic eruptions may occur.^7^ Metals like copper, nickel, cobalt, and chromium in these implants are the culprit in causing these skin reactions. Acute irritant contact dermatitis presents within minutes to hours after exposure to culprit chemical and is localized in nature. It commonly causes blisters, erythema, bulla, and oozing at the site of contact. Clinical findings in the presence of history of exposure to chemicals helps in diagnosis of irritant contact dermatitis. Acute onset of symptoms within minutes to hours after exposure to chemical points toward diagnosis of acute irritant contact dermatitis. There are reported cases of allergic reactions to endovascular, orthopedic, and dental metal implant. Removal of metallic implants in such cases results in resolution of symptoms in most cases.

PTFE used in prosthetic AV graft is considered to be chemically inert with minimal tendency to stimulate local or systemic inflammation. Hypersensitivity reactions to PTFE are infrequent though rare complications are increasingly being seen due to rising population of patients having AV grafts. Our patient presented with redness, swelling, and discharge at surgical site within hours after AV graft procedure. He was started on broad spectrum antibiotics for AV graft infection but blood and drainage cultures were consistently negative for bacteria. Exploration of AV graft was done but cultures were again negative. Due to acute onset of symptoms after graft creation, a diagnosis of AV graft contact dermatitis was made and decision was made to remove the graft. Graft removal resolved the symptoms in our patient. Diagnosis of contact dermatitis was challenging due to fever and elevated WBC at the time of admission.

## Conclusions

AV graft contact dermatitis is a rare complication that may mimic AV graft infection, which is a more common complication. It should be suspected if a patient’s symptoms continue to get worse despite broad-spectrum antibiotics and drainage cultures are consistently negative. Diagnosing contact dermatitis may be challenging in the presence of fever and elevated WBCs. Contact dermatitis can cause mild fever, leukocytosis, and elevated erythrocyte sedimentation rate if local inflammation is severe. Acute onset of symptoms within minutes to hours of AV graft placement may help in differentiating AV graft allergic reaction from AV graft infection.
